# Deep learning-based acceleration of high-resolution compressed sense MR imaging of the hip

**DOI:** 10.1016/j.ejro.2025.100656

**Published:** 2025-05-02

**Authors:** Alexander W. Marka, Felix Meurer, Vanessa Twardy, Markus Graf, Saba Ebrahimi Ardjomand, Kilian Weiss, Marcus R. Makowski, Alexandra S. Gersing, Dimitrios C. Karampinos, Jan Neumann, Klaus Woertler, Ingo J. Banke, Sarah C. Foreman

**Affiliations:** a Institute of Diagnostic and Interventional Radiology, School of Medicine and Health, TUM Klinikum, Technical University of Munich (TUM), Ismaninger Str. 22, Munich 81675, Germany; b Musculoskeletal Radiology Section, School of Medicine and Health, TUM Klinikum, Technical University of Munich, Ismaninger Str. 22, Munich 81675, Germany; c Clinic of Orthopedics and Sports Orthopedics, Klinikum rechts der Isar, Technical University of Munich, Ismaninger Strasse 22, Munich 81675, Germany; d Philips GmbH, Röntgenstrasse 22, Hamburg 22335, Germany; e Department of Radiology and Biomedical Imaging, University of California San Francisco, 505 Parnassus Ave, San Francisco, CA, 94143, USA; f Department of Neuroradiology, LMU University Hospital, LMU Munich, Marchioninistraße 13, 80337, Munich, Germany; g Kantonsspital Graubünden, KSGR, Loestrasse 170, 7000 Chur, Switzerland; h Department of Diagnostic and Interventional Neuroradiology, School of Medicine and Health, TUM Klinikum, Technical University of Munich (TUM), Ismaninger Str. 22, Munich 81675, Germany

**Keywords:** Hip, MRI, Deep learning, Compressed Sense

## Abstract

**Purpose:**

To evaluate a Compressed Sense Artificial Intelligence framework (CSAI) incorporating parallel imaging, compressed sense (CS), and deep learning for high-resolution MRI of the hip, comparing it with standard-resolution CS imaging.

**Methods:**

Thirty-two patients with femoroacetabular impingement syndrome underwent 3 T MRI scans. Coronal and sagittal intermediate-weighted TSE sequences with fat saturation were acquired using CS (0.6 ×0.8 mm resolution) and CSAI (0.3 ×0.4 mm resolution) protocols in comparable acquisition times (7:49 vs. 8:07 minutes for both planes). Two readers systematically assessed the depiction of the acetabular and femoral cartilage (in five cartilage zones), labrum, ligamentum capitis femoris, and bone using a five-point Likert scale. Diagnostic confidence and abnormality detection were recorded and analyzed using the Wilcoxon signed-rank test.

**Results:**

CSAI significantly improved the cartilage depiction across most cartilage zones compared to CS. Overall Likert scores were 4.0 ± 0.2 (CS) vs 4.2 ± 0.6 (CSAI) for reader 1 and 4.0 ± 0.2 (CS) vs 4.3 ± 0.6 (CSAI) for reader 2 (p ≤ 0.001). Diagnostic confidence increased from 3.5 ± 0.7 and 3.9 ± 0.6 (CS) to 4.0 ± 0.6 and 4.1 ± 0.7 (CSAI) for readers 1 and 2, respectively (p ≤ 0.001). More cartilage lesions were detected with CSAI, with significant improvements in diagnostic confidence in certain cartilage zones such as femoral zone C and D for both readers. Labrum and ligamentum capitis femoris depiction remained similar, while bone depiction was rated lower. No abnormalities detected in CS were missed in CSAI.

**Conclusion:**

CSAI provides high-resolution hip MR images with enhanced cartilage depiction without extending acquisition times, potentially enabling more precise hip cartilage assessment.

## Highlights

• CSAI framework enhances hip cartilage depiction in MRI scans.
• Higher resolution images achieved without extending scan times.
• Increased diagnostic confidence in detecting cartilage lesions.
• Comparable labrum and ligament depiction to standard CS.
• Potentially improves diagnostic accuracy and patient care in hip imaging.

## Graphical Abstract

**Figure:**
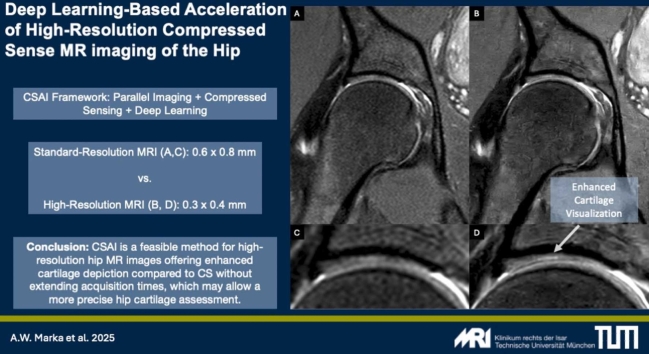

## 1 Introduction

Hip pain affects adults across diverse age groups and activity levels [1]. While a standing radiograph is often the initial imaging recommendation, magnetic resonance imaging (MRI) has become indispensable in modern musculoskeletal imaging. With superior soft tissue contrast and the absence of ionizing radiation, MRI is the preferred modality for diagnosing and managing hip pathologies, including labral tears, tendinopathies, and articular cartilage lesions affecting the acetabulum and femoral head [2], [3], [4], [5], [6]. Femoroacetabular impingement syndrome (FAIS) is known to cause early and severe cartilage damage [7]. Early osteoarthritis with the need of concomitant early hip replacement (THA) may result. Hip Arthroscopy with its modern opportunities of cartilage repair has gained vast importance, representing a valid joint-preservation tool conferring a 42 % relative risk reduction in the progression of osteoarthritis [8].

Depiction of the hyaline cartilage poses challenges due to the thin bony coverage, ranging from approximately 1 to 3 mm and the spherical shape of the femoral head, complicating spatial tissue characterization [9], [10], [11]. Therefore, high-resolution imaging is required for optimal visualization of cartilage lesions [9], [10], [11], [12]. However, the acquisition process of high-resolution sequences is inherently slow, attributed to extended encoding times, resulting in prolonged scans, elevated examination costs, and diminished patient throughput [13], [14]. Furthermore, maintaining stillness for several minutes, even for healthy individuals, proves challenging, and often compromising the image quality of high-resolution sequences with motion artifacts [15].

Advancements in MR imaging, such as parallel imaging (PI) and compressed sense (CS), have accelerated image acquisition. CS uses iterative reconstruction to minimize acquired k-space lines and reconstruct missing data [16], [17], while parallel imaging utilizes multiple coils to simultaneously acquire a reduced amount of k-space data [18], [19]. In the context of hip osteoarthritis, Kijowski et al.'s study showed that CS sequences provided more precise visualization of cartilage thickness compared to conventional three-dimensional Fast Spin Echo (3D FSE) sequences, improving the detection of early cartilage degeneration [20]. Recent implementations of deep learning(DL)-based methods have the potential to further reduce MR image acquisition times and optimize image quality [21], [22], [23], [24]. Previous studies have demonstrated the effectiveness of DL in accelerating imaging without compromising image quality or diagnostic performance, achieving significant scan time reductions in diverse body regions, including cardiac and musculoskeletal imaging [24], [25], [26], [27]. This comprehensive strategy has the potential to decrease scan time, improve motion-induced blurring, and enhance overall image clarity. To date, only one study by Herrmann et al. has demonstrated the feasibility of DL-based acceleration of two-dimensional Turbo Spin Echo (TSE) sequences with DL reconstruction for routine clinical hip MRI, revealing a significant reduction in acquisition time without compromising image quality [23]. However, to the best of our knowledge, the effectiveness of DL-based methods for acquiring high-resolution MR images of the hip has not been evaluated.

The aim of this study was therefore to evaluate a Compressed Sense Artificial Intelligence framework (CSAI) that combines PI, CS and DL-based artificial intelligence techniques for high-resolution imaging of the hip compared to standard-resolution CS imaging acquired in the same timeframe. The study compares image quality and diagnostic performance between both techniques.

## 2 Materials and methods

### 2.1 Subject selection

Patients were prospectively enrolled between January and May 2022. Prior to participation, all individuals provided informed consent, and the study received approval from the local institutional review board (BLINDED FOR REVIEW). All Patients were referred by our orthopedic hip preservation department with symptomatic FAIS prior to hip arthroscopy for FAIS treatment.

The diagnosis of FAIS was established based on a combination of clinical history, physical examination, and imaging findings in accordance with standard clinical guidelines [28]. Inclusion criteria were: (1) age between 18 and 65 years, (2) hip pain for more than 3 months, and (3) clinical signs of FAIS, including a positive flexion-adduction-internal rotation (FADIR) impingement test [28], [29]. Imaging confirmation of cam or pincer morphology was also required[29], [30], [31]. Patients who met these criteria underwent arthroscopic treatment within 3 months following MRI examination. Exclusion criteria included: (1) prior hip surgery, (2) advanced osteoarthritis (Tönnis angrade >1), (3) hip dysplasia, (4) inflammatory arthropathy, and (5) contraindications to MRI, such as the presence of pacemakers, other implanted electronic devices, or pregnancy.

### 2.2 Data acquisition

This study explores the effectiveness of a convolutional neural network (CNN), termed Adaptive-CS-Network (CSAI), which integrates and improves the conventional CS algorithm as presented by Pezzotti et al. [21]. Inspired by the iterative shrinkage-threshold algorithm (ISTA) presented by Zhang et al. [32], the Adaptive-CS-Network incorporates multiscale sparsification in a problem-specific learnable manner. It combines a CNN-based sparsifying approach with compressed sense for image reconstruction, ensuring data consistency and integrating domain-specific knowledge. The Adaptive-CS-Network replaces the wavelet transform with a CNN as a sparsifying transform in the compressed sense algorithm while preserving domain-specific knowledge and maintaining data consistency. Unlike Pezzotti’s model, the CNN used in this study was pre-trained on around 740,000 sparsifying MR images, covering both 1.5 T and 3 T images across various anatomies and contrasts. The algorithm has been optimized for execution on standard reconstruction hardware and has been productized in the meantime by the vendor (SmartSpeed, Philips Healthcare).

All examinations were performed on a 3 T MR scanner (Ingenia Elition; Philips Healthcare) using a combination of a 16-channel anterior coil and a 12-channel posterior coil integrated in the patient table. The examination protocol included the following 2D sequences: sagittal and coronal intermediate-weighted (IM) turbo spin echo (TSE) sequences with spectral presaturation with inversion recovery (SPIR) for fat saturation. All sequences were obtained two times: (1) with an acceleration factor of 1–1.2 reconstructed using CS and (2) with an acceleration factor of 2.3–3.3 reconstructed using CSAI. The CS protocol had a standard resolution of 0.6 × 0.8 mm. The CSAI protocol had a high resolution of 0.3 × 0.4 mm. Further details of the imaging protocols are shown in Table 1.

**Table 1:** Sequence parameters of the sequences acquired using CS and CSAI.

**Pulse sequence**	**sag PD FS TSE**		**cor PD FS TSE**	
	**CS**	**CSAI**	**CS**	**CSAI**
TR (ms)	2500	2500	1800	1800
TE (ms)	30	30	30	30
Echo Train Length (ETL)	11	10	10	10
Acq. Resolution (mm)	0.6 × 0.8	0.3 × 0.4	0.6 × 0.8	0.3 × 0.4
Slice Thickness (mm)	3	3	3	3
Field of View (mm)	160 × 160	160 × 160	190 × 170	190 × 170
Number of Slices	22	22	22	22
Signal Averages	1.5	2	1.5	2
Acceleration factor	1.2	3.3	1	2.3
Scan Time (minutes)	3:55	3:55	3:54	4:12

Note: TR: Repetition Time TE: Echo Time ETL: Echo Train Length Acq. Resolution: Acquisition Resolution sag PD FS TSE: Sagittal Proton Density Fat-Suppressed Turbo Spin Echo cor PD FS TSE: Coronal Proton Density Fat-Suppressed Turbo Spin Echo

Quantitative Image Analysis The signal-to-noise ratio (SNR) was calculated for fluid, muscle, and tendon using a single-image, region-of-interest (ROI)–based method. For each tissue, ROIs were manually placed in consistent anatomical locations across three consecutive slices. SNR was determined by dividing the mean signal intensity within the ROI by the standard deviation of signal intensity within the same ROI. This approach is commonly used in clinical research when repeated acquisitions or noise-only images are not available and has been recommended as a practical and reproducible method for evaluating image quality, particularly in advanced MR techniques such as compressed sensing and AI-based reconstructions [33]. Background-based SNR estimation was not used, as it has been shown to be inaccurate in systems employing phased-array coils and parallel imaging, where noise characteristics deviate from the assumptions required for traditional methods [33].

### 2.3 Semi-quantitative image analysis

**Fig. 1:**
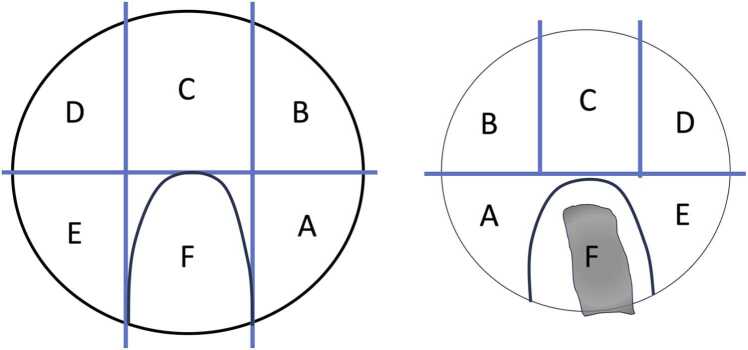
Diagram of acetabular (left) and femoral (right) cartilage Zones adapted from The Geographic Zone Method by Ilizaliturri et al. [34] modified by Griffin et al. [35], [36]. For right and left hip, the anterior-inferior zone is zone E, anterior-superior is zone D, (mid)-superior is zone C, posterior-superior is zone B, posterior-inferior is zone A, and mid-inferior is zone F. The head is presented dislocated in an “open-book fashion”; therefore the head is a “mirror image of the acetabulum.” Zone F on the left is the acetabular notch. Zone F on the right contains the ligamentum capitis femoris.

All MR images were independently evaluated by two readers: (1) a radiology resident (BLINDED FOR REVIEW, reader 1) with four years of experience and specific and dedicated training in musculoskeletal radiology by an expert musculoskeletal radiologist (BLINDED FOR REVIEW) ensuring a high level of expertise in evaluating the relevant imaging features, and (2) a fellowship-trained musculoskeletal radiologist (BLINDED FOR REVIEW, reader 2) with eight years of experience. A 4-week interval between readings of CS and CSAI datasets was maintained to mitigate recall bias. Readers were blinded to all clinical information and intraoperative findings. The readers assessed the depiction and presence of abnormalities of the ligamentum capitis femoris, the labrum, the acetabular and femoral cartilage, as well as the acetabular and femoral bone. The acetabular and femoral cartilage were divided into six zones (A-F) using the Geographic Zone Method by Ilizaliturri et al. [34] modified by Griffin et al. [35], [36], as depicted in Fig. 1. Zone F, corresponding to the acetabular fossa and ligamentum capitis femoris origin, was excluded from rating.

An ordinal 5-point Likert scale (ranging from 1 = poor to 5 = excellent) was used to assess the depiction of all anatomical structures, as described previously [24], including partial volume effect, blurring, discrimination from adjacent structures, and signal homogeneity. Abnormalities of the ligamentum capitis femoris were classified as 0, no pathology; 1, degenerative changes; 2, partial tear; and 3, complete tear. Labral abnormalities were graded as 0, no abnormality; 1, degeneration (abnormal signal); 2, intralabral tear (intralabral signal intensity extending to the labral surface); and 3, avulsion. Degenerative changes of the articular cartilage were graded as 0, normal; 1, abnormal signal; 2, superficial lesion (<50 % thickness); 3, deep lesion (>50 % thickness); and 4, osteochondral defect. In addition, diagnostic confidence for all detected abnormalities was recorded on a 5-point ordinal scale (ranging from 1 = poor to 5 = excellent).

### 2.4 Statistical analysis

The statistical analysis was performed with SPSS, version 29.0 (IBM) using a two-sided 0.05 level of significance (A.W.M.). CS served as the standard of reference for all statistical comparisons, given its current status as the prevailing imaging technique in our clinical practice. The Wilcoxon signed-rank test was used to evaluate distinctions in image quality, as well as the detection and classification of abnormalities, between the CS and CSAI protocols. Interobserver correlation of detected cartilage abnormalities were determined using Cohen’s kappa.

## 3 Results

### 3.1 Patient characteristics

A total of 32 subject were included (mean age, 37.5 years [range 21 – 61; Standard Deviation 11.7 years]; 8 women). The mean symptom duration before arthroscopic intervention was 31.7 ± 42.1 months. Details of patient characteristics are shown in Table 2.

**Table 2:** Patient characteristics.

**Patient Characteristics**	**N = 32**	**Percentages**
Age (years)*	37.5 (11.7)	
Female/Male	8/24	25/75
Weight (kg)*	81 (15.3)	
Height (cm)*	178 (9.7)	
Symptom duration (months)*	31.7 (42.1)	
Side of surgery (left/right)	19/13	59.4/40.6

* Data is given as mean ± standard deviation

Quantitative Image Analysis

**Fig. 2:**
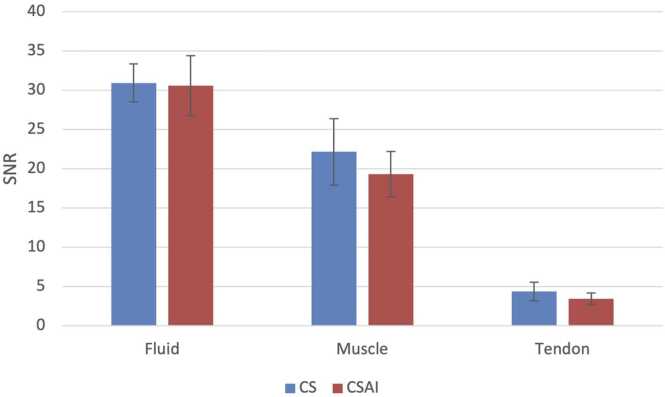
Signal-to-noise ratio (SNR) of fluid, muscle, and tendon for CS and CSAI reconstructions. Data are presented as mean ± standard deviation. No significant differences in SNR were observed between the two methods across all tissues.

SNR values for fluid, muscle, and tendon were calculated for both CS and CSAI sequences (Fig. 2). No significant differences were observed in SNR between the two reconstruction methods across all tissue types. For fluid, SNR was 30.9 ± 5.4 with CS and 30.6 ± 8.5 with CSAI. Muscle SNR measured 22.2 ± 9.5 for CS and 19.3 ± 6.4 for CSAI, while tendon SNR was 4.4 ± 2.6 and 3.4 ± 1.7 for CS and CSAI, respectively. These findings indicate comparable image quality in terms of signal-to-noise ratio between CS and CSAI.

#### 3.1.1 Depiction of anatomical structures

**Fig. 3:**
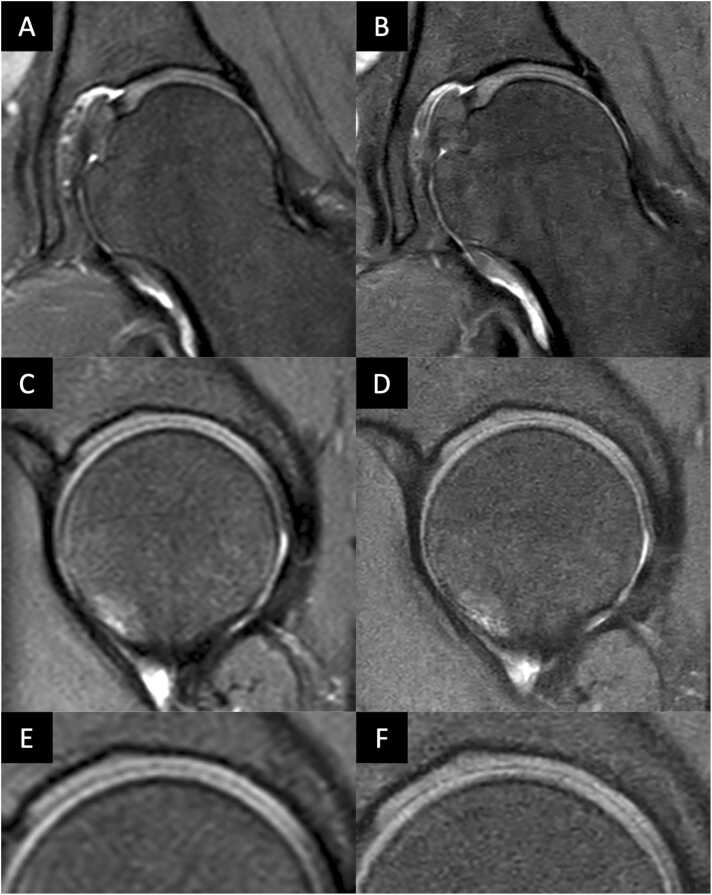
Coronal and sagittal conventional CS TSE images (A, C, E) and high-resolution IM-weighted CSAI TSE images (B, D, F) of a 31-year-old female patient. The images A and B are in the coronal plane, and C and D are in the sagittal plane with saggital zooms in E and F. Note the detailed depiction of the acetabular and femoral cartilage and the improved discrimination from adjacent structures in the high-resolution CSAI TSE image compared to standard-resolution CS TSE image.

**Fig. 4:**
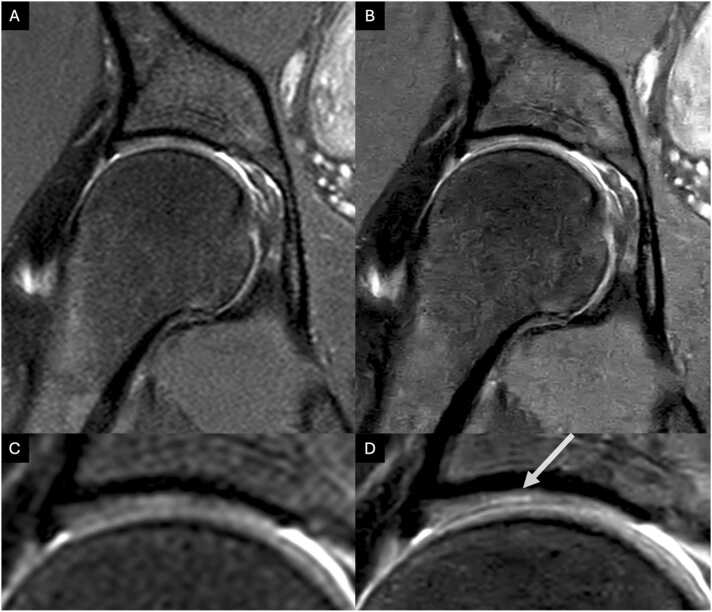
Coronal conventional CS TSE image (A, C) and high-resolution IM-weighted CSAI TSE image (B, D) of a 33-year-old female patient. The images C and D show a zoomed view of a hyperintensity in acetabular zone C, wich represents a partial thickness cartilage lesion measuring 5 mm (arrow). Note the detailed depiction of this defect in the high-resolution CSAI TSE image compared to the CS TSE image.

**Fig. 5:**
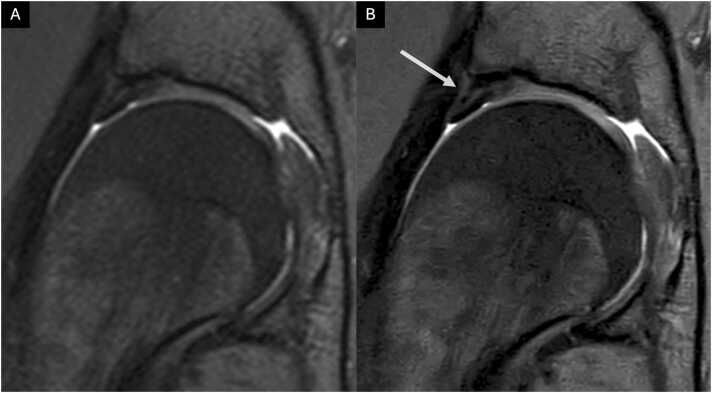
Coronal conventional CS TSE image (A) and high-resolution IM-weighted CSAI TSE image (B) of a 21-year-old female patient. Complex tear of the antero superior Labrum extending to the surface (arrow). Note the improved discrimination from adjacent structures and the detailed depiction of the labral substance in the high-resolution CSAI TSE image compared to the standard-resolution CS TSE image. Supplementary material S1: Cohen's Kappa for interrater reliability for standard resolution CS and high resolution CSAI.

Statistical analysis showed significant differences between the two sequences in the majority of the graded anatomical structures. Fig. 3 shows an example of CS vs. CSAI of a healthy hip. Both Readers graded the overall depiction acetabular and femoral cartilage significantly improved in CSAI compared to CS with a Likert score for overall cartilage depiction of 4.0 ± 0.2 (CS) vs 4.3 ± 0.6 (CSAI) for reader 1 and 4.0 ± 0.2 (CS) vs 4.2 ± 0.6 (CSAI) for reader 2 (both with p ≤ 0.001). Fig. 4 shows an image comparison of CS and CSAI for a cartilage defect. Furthermore Reader 1 graded the depiction of the Labrum significantly improved in CSAI with a score of 3.9 ± 0.4 (CS) and 4.3 ± 0.6 (CSAI) (p ≤ 0.05)*.* Fig. 5 shows an image comparison of CS and CSAI for a labral defect. Nevertheless, Reader 1 and 2 graded the bone structure of the femur to be significantly worse depicted in CSAI (p ≤ 0.05 and p ≤ 0.001, respectively). Reader 2 also observed significantly worse depiction of acetabular bone structure in CSAI (p ≤ 0.05). There was no statistical difference in the depiction of the ligamentum capitis femoris. Detailed information on all graded anatomical structures is shown in Table 3.

**Table 3:** Depiction of anatomical structures.

**Anatomical complex**	**Reader 1**		**Reader 2**	
	**CS**	**CSAI**	**CS**	**CSAI**
Ligamentum capitis femoris	3.7 ± 0.6	3.7 ± 0.7	3.8 ± 0.5	3.8 ± 0.7
Labrum	3.9 ± 0.4	4.3 ± 0.6*	4.1 ± 0.4	4.2 ± 0.5
Cartilage- Acetabulum				
Zone A	3.8 ± 0.4	4.2 ± 0.6*	4.0 ± 0.0	4.3 ± 0.6*
Zone B	3.9 ± 0.4	4.3 ± 0.6*	4.0 ± 0.0	4.3 ± 0.6*
Zone C	3.8 ± 0.4	4.3 ± 0.6*	4.0 ± 0.0	4.3 ± 0.6*
Zone D	3.8 ± 0.4	4.3 ± 0.7*	4.0 ± 0.0	4.3 ± 0.7*
Zone E	3.9 ± 0.4	4.3 ± 0.6*	4.0 ± 0.2	4.3 ± 0.6
Cartilage - Femur				
Zone A	3.9 ± 0.4	4.2 ± 0.6*	4.0 ± 0.2	4.3 ± 0.6*
Zone B	3.8 ± 0.5	4.2 ± 0.6*	3.9 ± 0.2	4.2 ± 0.6
Zone C	3.9 ± 0.2	4.4 ± 0.7*	3.9 ± 0.2	4.3 ± 0.7*
Zone D	3.8 ± 0.4	4.1 ± 0.6*	3.9 ± 0.3	4.0 ± 0.5
Zone E	3.7 ± 0.5	4.1 ± 0.6*	3.8 ± 0.4	4.1 ± 0.5*
Overall Cartilage	4.0 ± 0.2	4.3 ± 0.6**	4.0 ± 0.2	4.2 ± 0.6**
Bone				
Acetabulum	4.0 ± 0.6	3.8 ± 0.5	4.2 ± 0.5	3.9 ± 0.6*
Femur	4.1 ± 0.4	3.8 ± 0.6*	4.2 ± 0.4	3.8 ± 0.5**

Data are presented as means ± standard deviation

5-point Likert scale (5 = best; 1 = worst)

* *p* ≤ 0.05 using CS as standard of reference

** *p* ≤ 0.001 using CS as standard of reference

### 3.2 Assessment of abnormalities

Table 4 provides details on the assessment of cartilage abnormalities and diagnostic confidence. Both readers detected more abnormalities in high-resolution CSAI images compared to standard-resolution CS images (reader 1: n = 105/115 (CS/CSAI), reader 2: n = 110/112 (CS/CSAI). All abnormalities detected in standard-resolution CS images were also identified in the high-resolution CSAI images. The overall diagnostic confidence for cartilage was higher in CSAI. Reader 1 and Reader 2 reported diagnostic confidence levels of 3.5 ± 0.7 and 3.9 ± 0.6 for CS, while CSAI yielded higher confidence levels of 4.0 ± 0.6 and 4.1 ± 0.7 for Reader 1 and Reader 2, respectively (p ≤ 0.001). Moreover, the diagnostic confidence was significantly higher for abnormalities in femoral cartilage zones A, C, and D (p ≤ 0.05) for reader 2. For reader 1 the diagnostic confidence was significanlty higher for abnormalities in acetabular cartilage zones C and D (p ≤ 0.05) and femoral zones C and D (p ≤ 0.001 and p ≤ 0.05, respectively). No significant differences were found for the diagnostic confidence for detecting abnormalities of the labrum, the acetabular and femoral bone or the lig. capitis femoris, though only one abnormality was detected for the lig. capitis femoris by both readers. These results are provided in Table 5. Interreader correlation of detected cartilage abnormalities was good to excellent for femoral cartilage with *κ* = 0.67–1.0 in for CS sequences and *κ* = 0.71–1.0. However the correlation varied more in the acetabular carilage zones for both sequences with *κ* = 0.48–1.0. Cohen’s kappa calculated for each cartilage zone is shown in supplementary material S1.

**Table 4:** Detected cartilage abnormalities and diagnostic confidence.

		**Reader 1**				**Reader 2**			
**Anatomical complex**	**Grade**	**CS**		**CSAI**		**CS**		**CSAI**	
		***n***	**Rating**	***n***	**Rating**	**n**	**Rating**	**n**	**Rating**
Cartilage - Acetabulum									
Zone A	I								
	II	1		1		1		1	
	III					2		2	
	IV								
Total		1	3 ± n/a	1	3 ± n/a	3	3.3 ± 0.6	3	3.3 ± 0.6
Zone B	I			1					
	II	1		2					
	III	1		1		1		1	
	IV					1		1	
Total		2	3 ± 1.4	4	3.8 ± 1.0	2	4.0 ± 0.0	2	4.5 ± 0.7
Zone C	I	3		2		2		2	
	II	9		12		9		10	
	III	13		13		14		14	
	IV	2		2		3		2	
Total		27	3.7 ± 0.7	29	4.1 ± 0.7*	28	4.1 ± 0.4	28	4.3 ± 0.8
Zone D	I	4		4		4		4	
	II	4		2		4		3	
	III	10		13		10		11	
	IV	1		1		2		2	
Total		19	3.7 ± 0.7	20	4.3 ± 0.7*	19	4.1 ± 0.6	20	4.4 ± 0.7
Zone E	I								
	II								
	III					1		1	
	IV	1		1		1		1	
Total		1	3.0 ± n/a	1	4 ± n/a	2	4.0 ± 0.0	2	4.0 ± 0
Cartilage - Femur									
Zone A	I	2		2		2		1	
	II	6		7		4		5	
	III	6		6		8		8	
	IV	1		1		1		1	
Total		15	3.7 ± 0.7	16	4.1 ± 0.7	15	3.8 ± 0.6	15	4.1 ± 0.6*
Zone B	I	1		2		2		2	
	II								
	III	3		3		3		3	
	IV								
Total		4	2.8 ± 0.5	5	4.2 ± 0.4	5	3.4 ± 0.5	5	4.2 ± 0.4
Zone C	I	2		2		3		4	
	II	14		14		9		9	
	III	8		10		12		12	
	IV								
Total		24	3.3 ± 0.6	26	3.9 ± 0.4**	24	3.6 ± 0.6	25	3.8 ± 0.6*
Zone D	I	4		6		3		4	
	II	2		2		3		2	
	III	2		1		2		2	
	IV								
Total		8	3.3 ± 0.5	9	3.8 ± 0.4*	8	3.5 ± 0.5	8	4.0 ± 0.8*
Zone E	I								
	II	3		3		1		1	
	III	1		1		3		3	
	IV								
Total		4	3.3 ± 1.0	4	4.3 ± 0.5	4	3.5 ± 0.6	4	4.3 ± 0.5
Overall Cartilage Defects			3.5 ± 0.7		4.0 ± 0.6**		3.9 ± 0.6		4.1 ± 0.7**

Data are presented as means ± standard deviation

5-point Likert scale (5 = best; 1 = worst)

* *p* ≤ 0.05 using CS as standard of reference

** *p* ≤ 0.001 using CS as standard of reference

**Table 5:** Further detected abnormalities and diagnostic confidence.

		**Reader 1**				**Reader 2**			
**Anatomical complex**	**Grade**	**CS**		**CSAI**		**CS**		**CSAI**	
		***n***	**Rating**	***n***	**Rating**	**n**	**Rating**	**n**	**Rating**
Ligamentum capitis femoris	I	15		15		15		15	
	II	1		2		1		2	
	III								
Total		16	3.6 ± 0.8	17	3.6 ± 0.7	16	3.8 ± 0.7	17	3.7 ± 0.7
Labrum	I	8		8		10		10	
	II	16		16		16		16	
	III	6		6		5		5	
Total		30	4.1 ± 0.5	30	4.1 ± 0.6	31	4.3 ± 0.5	31	4.1 ± 0.5
Bone - Acetabulum									
Subchondral Zysts		8	4.3 ± 0.7	8	4.4 ± 0.7	8	4.4 ± 0.7	8	4.4 ± 0.7
Bone Marrow Edema		13	4.4 ± 0.7	13	4.3 ± 0.8	11	4.5 ± 0.5	11	4.4 ± 0.8
Bone - Femur									
Subchondral cysts		1	3 ± n/a	1	5 ± n/a	1	5 ± n/a	1	5 ± n/a
Bone Marrow Edema		2	4.0 ± 1.4	1	4 ± n/a	1	5 ± n/a	0	n/a

Data are presented as means ± standard deviation

5-point Likert scale (5 = best; 1 = worst)

*p ≤ 0.05 using CS as standard of reference

^**^p ≤ 0.001 using CS as standard of reference

## 4 Discussion

In this study, we implemented an artificial intelligence framework to acquire high-resolution hip MRI images within the same time frame as standard-resolution images. This approach significantly improved the depiction of acetabular and femoral cartilage compared to standard-resolution conventional CS. CSAI identified more cartilage abnormalities with greater diagnostic confidence compared to CS. Although both readers noted slightly more lesions in CSAI than in CS, the overall defect count did not differ significantly. Notably, CSAI achieved these improvements within a similar time frame to the standard-resolution protocol, suggesting its practicality for clinical use.

Previous studies, such as Chaudhari et al. [37], utilized DL, specifically convolutional neural networks (CNNs), to generate high-resolution knee MR images from low-resolution input slices, demonstrating improved image quality. Similarly, Hermann et al. [23] compared a standard knee MRI protocol using generalized autocalibrating partial parallel acquisition reconstruction (GRAPPA) with a protocol involving undersampled MR data and DL-based reconstruction, revealing significantly better overall image quality in the latter without notable differences in anatomical structures and pathologies. Another study by Koch et al. [38] compared two denoising settings (50 % and 75 %) of a DL reconstruction model with conventional MRI reconstructions in orthopedic imaging, showing improved edge sharpness, signal-to-noise ratio, and contrast-to-noise ratio with strong radiologist preference for DL reconstructions. In our study, the CSAI sequence significantly enhanced image quality with a minimal increase in acquisition time, detecting the same abnormalities as the conventional sequence without a significant difference in recorded diagnostic confidence. These findings suggest that implementing CSAI protocols in clinical practice could improve diagnostic image quality, though further validation studies are necessary to confirm these results.

Sutter et al. highlighted recent findings demonstrating superior diagnostic accuracy of MR arthrography over native MR imaging for assessing labral pathologies and acetabular cartilage conditions [39], [40]. Studies indicate potential improvement in detecting cartilage lesions with leg traction during MR arthrography, but additional clinical research is necessary to evaluate its added clinical value [6], [39]. Despite its effectiveness, MR arthrography can cause pain and side effects (e.g., pressure, headache, muscle ache, infection), which may discourage patients from undergoing this procedure [41]. Therefore, our study emphasizes the importance of enhancing image resolution in native and non-traction imaging, addressing concerns associated with MR arthrography.

Notably in our study the diagnostic confidence was highest for chondral abnormalities in zones C and D representing the superior and anterosuperior zones of the acetabulum as well as the corresponding articulating femoral zones. These zones are of most interest regarding diagnostic accuracy as they represent the typical zones for the vast majority of chondral damages in FAIS and concomitant cartilage repair [35], [42].

While the high resolution CSAI images showed a significant overall improvement over CS images, interestingly bone marrow appearance was rated worse in CSAI. This can be due to multiple reasons. The bone marrow signal is generally low in fat suppressed sequences, as were investigated in the current work. In combination with the high resolution and high acceleration factors as used in the CSAI acquisitions and given the little anatomical detail within in the bone marrow, the bone structures may not be fully denoised and might create an artificial appearance of the bone marrow for CSAI scans, while the noise in the bone marrow in CS scans might appear more homogeneous.

Despite yielding promising results, this study has some limitations that warrant attention in subsequent research. The absence of an external reference standard, such as arthroscopy, hinders the validation of diagnosed hip pathologies. The study's small sample size of 30 subjects, encompassing a limited range of pathologies, necessitates further investigation with a larger and more diverse cohort. While the primary goal was to validate results using an internal dataset, additional studies, encompassing temporal and external validation, are imperative. To integrate CSAI into clinical workflows effectively, it might be advantageous to introduce these imaging sequences alongside standard protocols, facilitating radiologists' comprehension and strategic implementation of CSAI.

In summary, the study demonstrates that the CSAI framework is an effective method for acquiring high-resolution MRI images of the hip. CSAI enhances the depiction of cartilage compared to standard-resolution sequences without increasing acquisition times. While this improvement in cartilage visualization and diagnostic confidence suggests that CSAI could be a valuable tool for high-resolution hip imaging, it is important to note that the lack of a surgical or arthroscopic gold standard limits the ability to definitively conclude a clinical benefit. Further studies incorporating surgical validation are needed to confirm the potential impact on diagnostic accuracy and patient care.

## Funding

The authors state that this work has not received any funding.

## Ethical Statement

Written informed consent was obtained from all subjects (patients) in this study.

Institutional Review Board approval was obtained.

## CRediT authorship contribution statement

**Weiss Kilian:** Writing – review & editing, Software, Conceptualization. **Ebrahimi Ardjomand Saba:** Visualization, Investigation. **Graf Markus:** Writing – review & editing, Investigation. **Twardy Vanessa:** Writing – review & editing, Data curation. **Foreman Sarah C.:** Writing – review & editing, Visualization, Methodology, Investigation, Conceptualization. **Meurer Felix:** Investigation, Formal analysis. **Banke Ingo J.:** Writing – review & editing, Investigation. **Marka Alexander Wolfgang:** Writing – original draft, Methodology, Investigation, Data curation, Conceptualization. **Woertler Klaus:** Writing – review & editing, Validation, Supervision. **Neumann Jan:** Writing – review & editing, Conceptualization. **Karampinos Dimitrios C.:** Writing – review & editing, Software, Conceptualization. **Gersing Alexandra S.:** Validation, Methodology. **Makowski Marcus R.:** Supervision, Resources.

## Declaration of Competing Interest

Co-author Kilian Weiss was employed by Philips GmbH Market DACH and Dimitrios C. Karampinos reports a relationship with Philips Healthcare that includes funding grants; but both were not involved in handling or analysis of data. The other authors declare that they have no known competing financial interests or personal relationships that could have appeared to influence the work reported in this paper.
