# Supplementary material for: Deep learning-based acceleration of high-resolution compressed sense MR imaging of the hip
Source: Eur J Radiol Open. 2025 May 2;14:100656. doi: 10.1016/j.ejro.2025.100656 (PMC12123326; doi:10.1016/j.ejro.2025.100656)
Supplement: Supplementary file 1 — Supplementary material [file mmc1.docx]

Supplementary material S1: Cohen's Kappa for interrater reliability for standard resolution CS and high resolution CSAI.

|  | **Cartilage** | **CS** | **CSAI** |
| --- | --- | --- | --- |
| **Acetabulum** | Zone A | 0.48 | 0.48 |
|  | Zone B | 1 | 0.64 |
|  | Zone C | 0.61 | 0.84 |
|  | Zone D | 0.80 | 0.87 |
|  | Zone E | 0.65 | 0.65 |
| **Femur** | Zone A | 0.87 | 0.94 |
|  | Zone B | 0.87 | 1 |
|  | Zone C | 0.83 | 0.71 |
|  | Zone D | 0.67 | 0.76 |
|  | Zone E | 1 | 1 |
